# Neural Correlates of Virtual Reality Intervention in Children With Attention‐Deficit/Hyperactivity Disorder: A Resting‐State fMRI Study Based on Percent Amplitude of Fluctuation

**DOI:** 10.1002/brb3.71518

**Published:** 2026-05-29

**Authors:** Xinjie Yu, Jieling Zhu, Jiujiu Yang, Hongtao Hou, Guoqun Mao, Luhan Tang, Fuquan Wei

**Affiliations:** ^1^ Department of Radiology Tongde Hospital of Zhejiang Province Hangzhou Zhejiang Province China; ^2^ Department of Psychology Tongde Hospital of Zhejiang Province Hangzhou Zhejiang Province China

**Keywords:** attention‐deficit/hyperactivity disorder (ADHD), percent amplitude of fluctuation (PerAF), resting‐state fMRI (rs‐fMRI), virtual reality (VR)

## Abstract

**Objective:**

To investigate neural mechanisms associated with improvements in core symptoms of attention‐deficit/hyperactivity disorder (ADHD) following a virtual reality (VR) intervention, using resting‐state fMRI (rs‐fMRI) with the percent amplitude of fluctuation (PerAF) and standardized clinical behavioral assessments.

**Methods:**

Fifteen children with ADHD and fourteen age‐ and sex‐matched typically developing children were enrolled. A pre‐post intervention design was applied. Children with ADHD completed the Swanson, Nolan, and Pelham Rating scale‐fourth edition (SNAP‐IV), the matching familiar figures test (MFFT), and rs‐fMRI scans before and after the VR intervention. Healthy controls underwent identical assessments at baseline. Voxel‐wise analyses were conducted, and correlation analyses were conducted between changes in PerAF values and behavioral measures.

**Results:**

Following the VR intervention, children with ADHD showed significant reductions in SNAP‐IV scores for inattention, hyperactivity/impulsivity, and oppositional defiant behaviors, as well as fewer MFFT errors (P < 0.05). Compared with baseline, decreased PerAF values were observed in the right middle frontal gyrus, whereas increased PerAF values were found in the left paracentral lobule (PCL). Relative to healthy controls, the ADHD group exhibited elevated PerAF values in the left inferior temporal gyrus (ITG) after the intervention, which were positively correlated with oppositional defiant symptoms (r = 0.610).

**Conclusion:**

The VR intervention may ameliorate hyperactivity‐impulsivity and behavioral problems in children with ADHD by modulating neural activity in brain regions associated with cognitive control (right MFG) and sensorimotor integration (left PCL). However, its regulatory effects on core attentional deficits and temporo‐limbic circuits (left ITG) appear to be relatively limited. These findings provide preliminary neuroimaging evidence supporting the potential of VR as an adjunctive intervention for ADHD.

## Graphical abstract

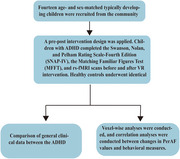

## 1 Introduction

Attention‐deficit/hyperactivity disorder (ADHD) is a prevalent neurodevelopmental disorder characterized by persistent inattention, hyperactivity, and impulsivity. The global prevalence of ADHD in children is estimated to be approximately 5–7% (Sayal et al. 2018), and affected individuals frequently experience learning difficulties and emotional‐behavioral problems that substantially impair academic performance, social functioning, and long‐term mental health outcomes (Bonham et al. 2021). Although psychostimulant medications remain the first‐line treatment and effectively alleviate core symptoms (León‐Barriera et al. 2023), their clinical utility is often constrained by adverse effects, poor adherence, and limited efficacy in addressing comorbid behavioral problems. Consequently, the development of safe, effective, and evidence‐based non‐pharmacological interventions has become an important focus in ADHD research.

In recent years, non‐pharmacological approaches such as behavioral therapy and cognitive training have shown promising clinical potential (Wolraich et al. 2019). Behavioral interventions can enhance attentional regulation and emotional control in children with ADHD (Feldman et al. 2018; Shrestha et al. 2020). However, they typically require extensive coordination among families and schools, involve long intervention periods, and incur relatively high costs. Virtual reality (VR) technology offers a novel paradigm for cognitive‐behavioral training by providing immersive and controllable simulated environments. Accumulating evidence indicates that VR‐based interventions yield beneficial effects in populations such as children with autism spectrum disorder (Yan et al. 2025) and individuals with post‐traumatic stress disorder (McLay et al. 2014). Owing to its high ecological validity and strong engagement, VR may be particularly suitable for pediatric interventions. Preliminary studies suggest that VR‐based training targeting attention and executive functions can improve behavioral performance in children with ADHD (Goharinejad et al. 2022; Zangiacomi et al. 2022); however, the neural mechanisms underlying these effects remain largely unclear, limiting the standardization and optimization of VR‐based interventions.

Resting‐state fMRI (rs‐fMRI) has been widely used to characterize intrinsic brain abnormalities in ADHD. Prior studies have reported alterations using multiple analytical frameworks, including amplitude‐based metrics such as ALFF/fALFF, local synchrony measures such as ReHo, graph‐based measures such as degree centrality, and functional connectivity analyses, suggesting distributed abnormalities across executive‐control, sensorimotor, and default‐mode systems in ADHD (Wang et al. 2017; Zhou et al. 2019). However, findings across cohorts have shown considerable heterogeneity, and relatively few studies have examined longitudinal changes in intrinsic brain activity following non‐pharmacological intervention in pediatric ADHD. PerAF, a voxel‐wise metric that expresses BOLD fluctuation amplitude as a percentage of mean signal intensity, has been proposed as a scale‐independent and relatively reliable measure of local spontaneous activity, which may complement previously used rs‐fMRI indices (Jia et al. 2020). Although VR‐based interventions have shown promising behavioral effects in children with ADHD (Wiguna et al. 2020), neuroimaging evidence‐particularly rsfMRI evidence regarding intervention‐related changes in intrinsic brain activity‐remains limited, and the neural mechanisms underlying potential benefits are still insufficiently understood (Jiang et al. 2022).

Therefore, the present study combined rs‐fMRI with PerAF and standardized clinical behavioral assessments, using both case‐control and within‐subject pre‐post designs, to identify ADHD specific patterns of brain functional modulation associated with VR intervention and to provide evidence at the neural mechanism level supporting its potential clinical application.

## 2 Materials and Methods

### 2.1 Participants

This study was approved by the Ethics Committee of our institution (Approval No. 2023‐013‐JY). Fifteen children with ADHD were consecutively recruited from the outpatient psychiatry clinic of our hospital between January 2023 and January 2024. ADHD diagnoses were established according to DSM‐5. Fourteen age‐ and sex‐matched typically developing children were recruited from the community as healthy controls (HC group).

The inclusion criteria for the ADHD group were as follows:

1. Fulfillment of DSM‐5 diagnostic criteria for ADHD.
2. Age between 6 and 14 years.
3. No prior treatment related to ADHD.
4. Full‐scale intelligence quotient (IQ) ≥ 80 as assessed by the Wechsler Intelligence Scale for Children.
5. No psychotropic medication use within the preceding three months.
6. Right‐handedness.

The inclusion criteria for the HC group were:

1. No history of psychiatric or neurological disorders.
2. Age between 6 and 14 years.
3. Full‐scale IQ ≥ 80.
4. No medication use within the preceding three months.
5. Right‐handedness.

Exclusion criteria for all participants included: presence of organic mental disorders, schizophrenia, bipolar disorder, personality disorders, or other major psychiatric illnesses; history of severe physical disease; contraindications to MRI scanning (e.g., metallic implants); family history of psychiatric disorders; and hearing or visual impairments that could interfere with task completion.

### 2.2 Clinical Behavioral Assessments

#### 2.2.1 Swanson, Nolan, and Pelham Rating Scale‐Fourth Edition (SNAP‐IV)

The SNAP‐IV is a widely used rating scale for assessing ADHD‐related symptoms, including inattention, hyperactivity/impulsivity, and oppositional behaviors (Costa et al. 2019). The parent‐rated version of the SNAP‐IV was used to assess ADHD‐related behavioral symptoms. The scale consists of 26 items rated on a 4‐point Likert scale (0 = “not at all,” 3 = “very much”). Items are grouped into three subscales: inattention (items 1–9), hyperactivity/impulsivity (items 10–18), and oppositional defiant behaviors (items 19–26). Subscale scores were calculated as the mean item score, with higher scores indicating greater symptom severity.

#### 2.2.2 Matching Familiar Figures Test (MFFT)

The MFFT is a commonly used neuropsychological task indexing reflection‐impulsivity and response style, with prior work supporting its validity and reliability for behavioral assessment (Viator et al. 2022). The MFFT was administered to assess impulsivity. The task consists of 20 trials, each presenting a target image alongside six similar images. Children were instructed to select the image identical to the target. The total number of errors was recorded and used as an index of impulsivity.

### 2.3 Virtual Reality Intervention

The VR training program was designed to specifically target sustained attention, visual selective attention, and behavioral response inhibition in children with ADHD. Training was conducted in an immersive virtual cave environment, in which participants controlled a firefly character from a third‐person perspective. By turning their head and focusing their gaze, children navigated the firefly to avoid randomly appearing obstacles and collect target objects.

Each training session incorporated multiple response inhibition task modules. Task difficulty was adaptively adjusted in real time based on individual performance, following a personalized and progressive training principle. Task difficulty was adaptively adjusted according to the child's performance in the preceding block/session, including response accuracy, reaction time, and commission/omission errors. The frequency and density of obstacles are dynamically adjusted based on individual performance. The difficulty level begins with a single obstacle appearing twice per minute, and at the most challenging level, ten obstacles each appear 10 times per minute. Each intervention session lasted approximately 30 min and was conducted once per week over a total period of 16 weeks.

### 2.4 MRI Data Acquisition

All participants underwent MRI scanning within two days after completing the clinical assessments, using a 3.0 Tesla MRI scanner (Magnetom Verio, Siemens, Germany) equipped with a 32‐channel head coil. Earplugs and foam padding were used to reduce scanner noise and minimize head motion.

EPI: Slices = 30; TR = 2000 ms; TE = 30 ms; flip angle = 90°; matrix size = 64 × 64; FOV = 220 × 220 mm^2^; slice thickness/gap = 4/0 mm; voxel size = 3.4 × 3.4 × 4.0 mm^3^; total scan duration = 6 min 46 s.

3D T1WI SPGR: Slices = 176; TR = 1900 ms; TE = 2.5 ms; flip angle = 9°; matrix size = 256 × 256; FOV = 250 × 250 mm^2^; slice thickness/gap = 1/0 mm; voxel size = 1.0 × 1.0 × 1.0 mm^3^; scan duration = 4 min 18 s.

### 2.5 Rs‐fMRI Data Preprocessing

Raw imaging data were first visually inspected using MRIcro software to exclude incomplete datasets or images with obvious artifacts. Subsequent preprocessing was performed using Matlab R2017a.

The preprocessing steps included:

1. Conversion of DICOM images to NIFTI format.
2. Removal of the first 10 volumes to allow for magnetic field stabilization.
3. Correction for head motion using the Friston 24‐parameter model, with exclusion criteria of head translation > 3 mm or rotation > 3°.
4. Spatial normalization to the standard EPI template.
5. Spatial smoothing with a 6 mm FWHM Gaussian kernel.
6. Regression of nuisance signals from white matter and cerebrospinal fluid.

### 2.6 PerAF Calculation

PerAF values were calculated using the RESTplus toolbox. The square root of the power spectrum was obtained, followed by bandpass filtering (0.01–0.08 Hz) to retain only low‐frequency components. For each voxel, the BOLD signal intensity at each time point was divided by the mean BOLD signal intensity across the entire time series, and the percentage value was computed. The averaged percentage across time points was used to generate whole‐brain PerAF maps for each participant.

### 2.7 Statistical Analysis

Statistical analyses were performed using IBM SPSS Statistics for Windows (version 26.0; IBM Corp., Armonk, NY, USA). Continuous variables with normal distributions are presented as mean ± standard deviation, while non‐normally distributed data are expressed as median (interquartile range). Between‐group comparisons were conducted using independent‐samples t‐tests or Mann–Whitney U‐tests, and within‐group comparisons were performed using paired‐samples t‐tests or Wilcoxon signed‐rank tests, as appropriate. Categorical variables were analyzed using chi‐square tests. Voxel‐wise group comparisons of PerAF were corrected for multiple comparisons using Gaussian random field (GRF) theory, with a voxel‐level threshold of P< 0.01 and a cluster‐level threshold of P < 0.05. Mean PerAF values were extracted from brain regions showing significant differences and correlated with behavioral scores using Pearson correlation analysis. False discovery rate (FDR) correction was applied to control for multiple comparisons.

## 3 Results

### 3.1 Clinical and Behavioral Results

No significant differences were observed between the ADHD and HC groups in age or sex distribution (P > 0.05), indicating comparable baseline characteristics.

**FIGURE 1:**
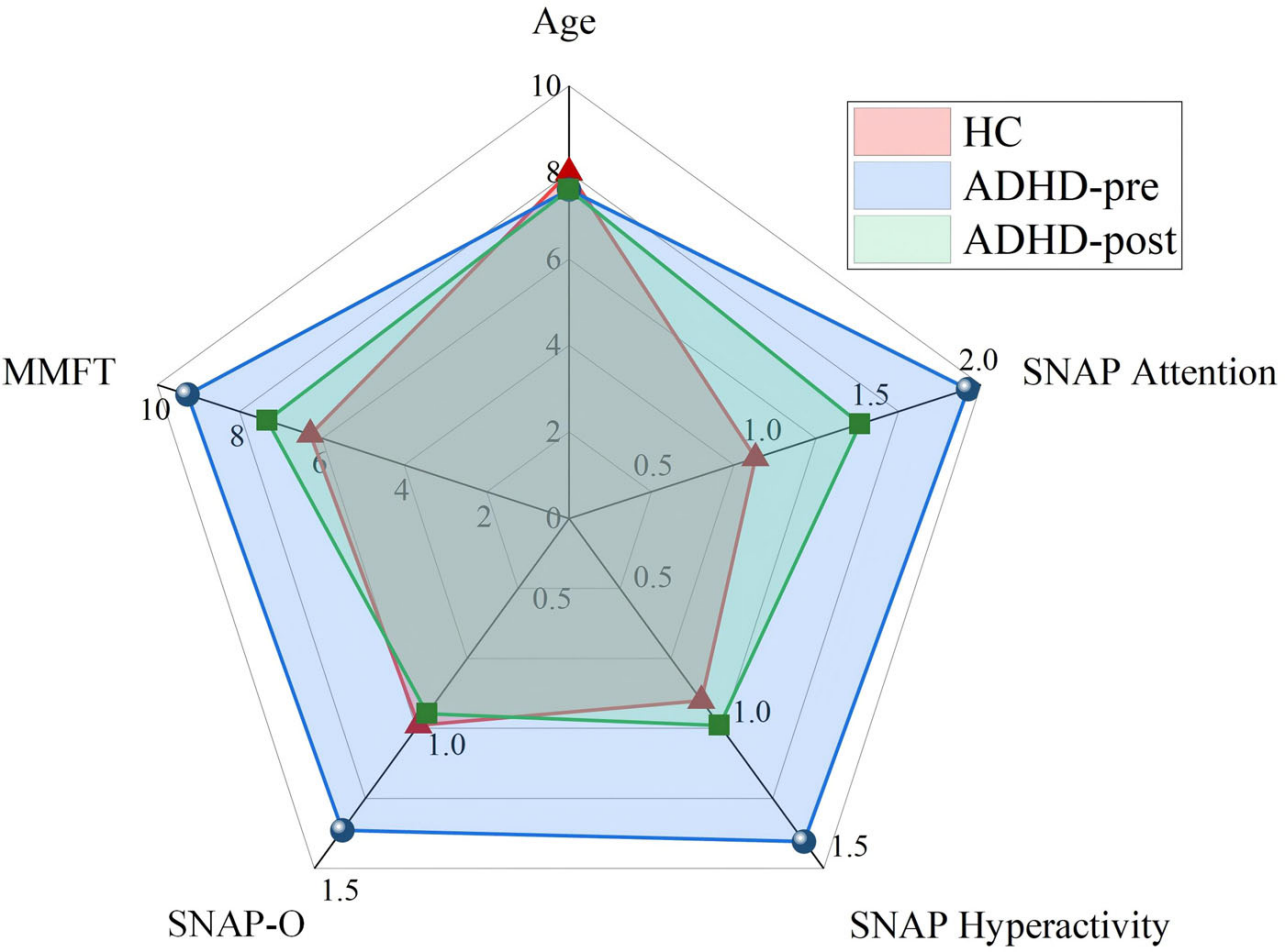
Comparisons of SNAP‐IV rating scale scores and age among groups. ADHD‐pre:before the VR intervention;ADHD‐post:after VR intervention.

At baseline, children with ADHD showed significantly higher scores across all clinical measures compared with the HC group (P < 0.01). After 16 sessions of VR intervention, the ADHD group demonstrated significant reductions in SNAP‐IV scores for inattention, hyperactivity/impulsivity, and oppositional defiant behaviors, as well as a significant decrease in MFFT error numbers (all P < 0.05). Following intervention, inattention scores in the ADHD group remained significantly higher than those of the HC group (P < 0.001), whereas no significant between‐group differences were found for hyperactivity/impulsivity, oppositional defiant behaviors, or MFFT performance (P > 0.05) (Table 1; Figure 1).

**TABLE 1:** Comparison of general clinical data between the ADHD and HC groups.

—	ADHD‐pre	ADHD‐post	HC	Intra‐ and inter‐group experiments		
				ADHD‐pre vs. ADHD‐post	ADHD‐pre vs. HC	ADHD‐post vs. HC
Age	7.60 ± 0.828	—	8.00 ± 0.961	—	*p* = 0.239; *t* = −1.203	—
Gender (male/female)	10/5	—	10/4	—	*p* = 0.782; *t* = 0.771	—
SNAP Attention	1.94 ± 0.324	1.442 ± 0.342	0.960 ± 0.157	*P* < 0.001*; *t* = 2.105	*P* < 0.001*; *t* = 10.5	*P* < 0.001*;*t*=4.816
SNAP Hyperactivity	1.40 ± 0.613	0.969 ± 0.352	0.876 ± 0.159	*p* = 0.027*; *Tt* = 2.054	*p* = 0.006*; *t* = 3.206	*p* = 0.373; *t* = 1.211
SNAP‐O	1.369 ± 0.362	0.969 ± 0.352	1.009 ± 0.160	*P* < 0.001*; *t* = 4.535	*p* = 0.002*; *t* = 3.506	*p* = 0.069; *t* = −1.892
MMFT	9.267 ± 2.576	7.333 ± 2.059	6.286 ± 1.816	*p* = 0.031*; *t* = 2.045	*P* < 0.001*; *tSNAP‐O* = 3.577	*p* = 0.157; *t* = 1.556
Framewise displacement(mm)	2.29 ± 0.366	2.40 ± 0.341	2.31 ± 0.346	*p* = 0.211; *t* = −1.317	*p* = 0.962; *t* = −0.052	*p* = 0.398; *t* = 0.859

Abbreviations:ADHD‐pre, before the VR intervention;ADHD‐post, after VR intervention;SNAO ‐ O, SNAP‐IV Oppositional Defiant scores.

*Note: P* < 0.05 indicates statistical significance. Values marked with * indicate statistically significant differences.

### 3.2 Neuroimaging Results

#### 3.2.1 Pre‐Intervention PerAF Differences Between ADHD and HC Groups

**FIGURE 2:**
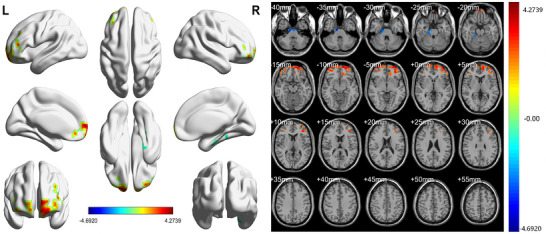
Group differences in PerAF values between the ADHD‐pre and HC groups. The left MFG and the right orbital MFG showed higher PerAF values, while the left cerebellar lobule VIII showed lower PerAF values.

Compared with healthy controls, children with ADHD exhibited significantly increased PerAF values in the left middle frontal gyrus (MFG) and the right orbital MFG, along with significantly decreased PerAF values in the left cerebellar lobule VI (Table 2; Figure 2).

**TABLE 2:** The differences in PerAF values among different brain regions in groups.

PerAF values	Brain regions	Voxels	MNI coordinates			T‐value
			*X*	*Y*	*Z*	
ADHD‐pre < HC	The left cerebellar lobule VIII	76	12	−12	−36	−4.692
ADHD‐pre > HC	The left MFG/ the right orbital MFG	87/63	−45	54	0	4.2739
ADHD‐post < ADHD‐pre	The right MFG	170	33	42	18	−7.6558
ADHD‐post > ADHD‐pre	The left PCL	82	−6	−33	72	4.581
ADHD‐post > HC	The left ITG	83	−48	−39	−9	4.9336

Abbreviations:ADHD‐pre, before the VR intervention;ADHD‐post, after VR intervention.

#### 3.2.2 Pre–Post Changes in PerAF Following VR Intervention

**FIGURE 3:**
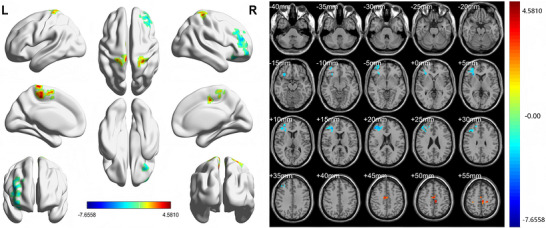
Group differences in PerAF values between the ADHD‐pre and ADHD‐post groups. The right MFG showed lower PerAF values, while the left PCL showed higher PerAF values.

Relative to baseline, children with ADHD showed a significant decrease in PerAF values in the right MFG and a significant increase in the left paracentral lobule (PCL) following VR intervention (*p* < 0.05, GRF‐corrected) (Table 2; Figure 3).

#### 3.2.3 Post‐Intervention PerAF Differences between ADHD and HC Groups

**FIGURE 4:**
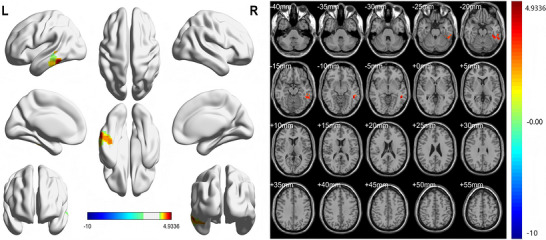
Group differences in PerAF values between the ADHD‐post and HC groups. The left ITG showed higher PerAF values.

After intervention, PerAF values in the left inferior temporal gyrus (ITG) remained significantly higher in the ADHD group compared with the HC group (*P*< 0.05, GRF‐corrected) (Table 2; Figure 4).

#### 3.2.4 Brain–Behavior Correlation Analysis

**FIGURE 5:**
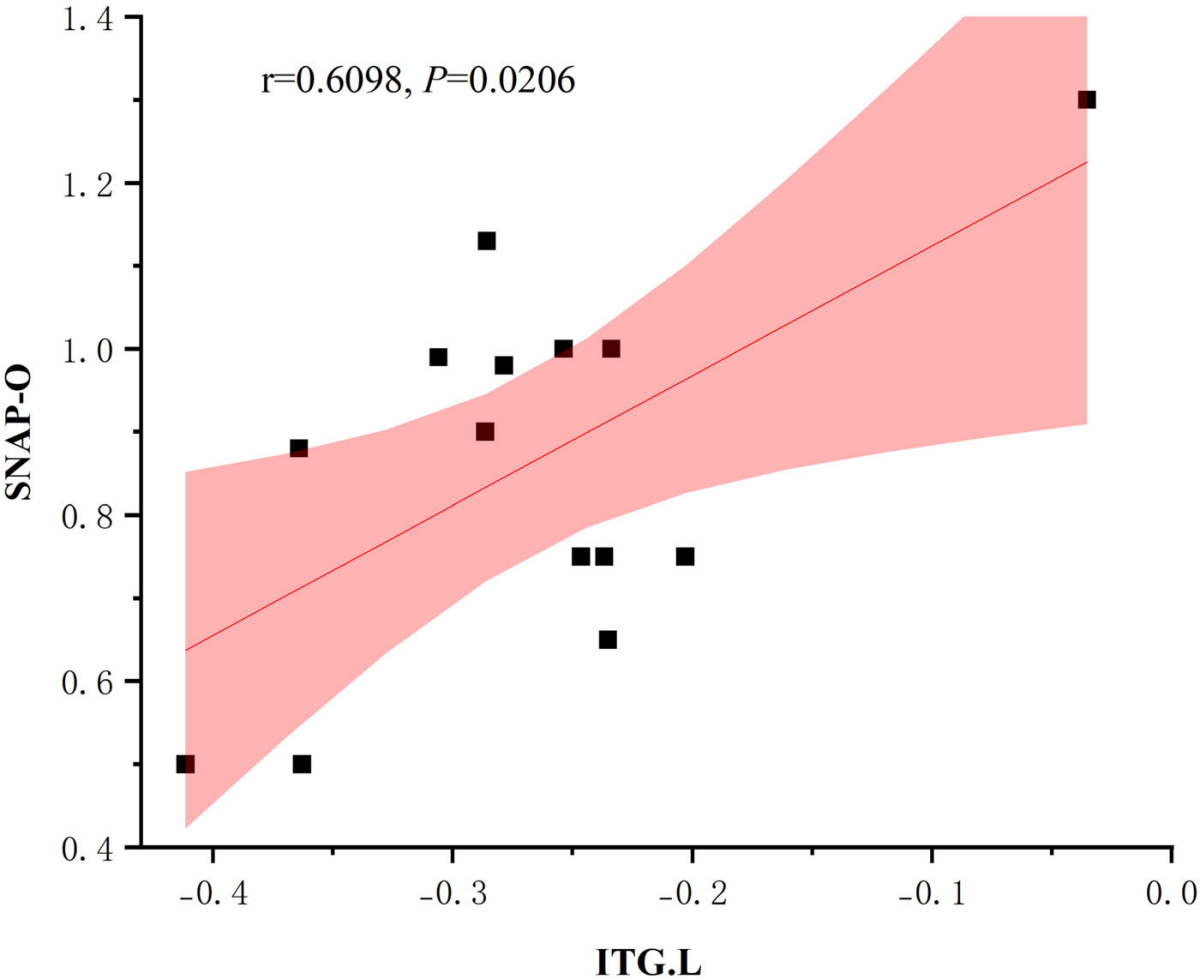
ITG.L: the left ITG. The figure included 14 data points because one point was excluded from the correlation analysis due to excessive head motion(head translation > 3 mm). PerAF values in the left ITG were positively correlated with SNAP‐IV oppositional defiant scores in children with ADHD.

Following VR intervention, PerAF values in the left ITG were positively correlated with SNAP‐IV oppositional defiant scores in children with ADHD (*r* = 0.610, *P* < 0.05, FDR‐corrected) (Figure 5).

## 4 Discussion

The present study employed PerAF to examine the effects of VR intervention on intrinsic brain activity in children with ADHD. The main findings can be summarized as follows: VR intervention was associated with significant improvements in hyperactivity/impulsivity and oppositional behaviors; following intervention, neural activity in the right MFG, a region implicated in cognitive control, showed a trend toward normalization, as reflected by decreased PerAF values, while activity in the left PCL, associated with sensorimotor integration, was increased; improvements in inattention were relatively limited, and elevated activity in the left ITG persisted after intervention and was positively correlated with oppositional defiant symptoms. Together, these findings suggest symptom‐domain specificity of VR intervention effects and corresponding specificity in the underlying neural circuits.

At baseline, children with ADHD exhibited increased PerAF values in the left MFG and orbital MFG, which is consistent with previous evidence indicating dysfunction within frontal executive control networks in ADHD. Longitudinal studies by Shaw et al. have demonstrated close associations between cortical thickness trajectories in frontal regions and hyperactivity symptoms (Shaw et al. 2013; Norman et al. 2016; Hoogman et al. 2019). Further work by Edmund and colleagues highlighted the relevance of orbitofrontal cortical development and its interactions with the ventral striatum and limbic system in cognitive and emotional symptomatology (Rolls 2023). The MFG, particularly the dorsolateral prefrontal cortex, plays a central role in attentional allocation, conflict monitoring, and impulse inhibition. Hyperactivation in this region may reflect reduced neural efficiency or compensatory effort during cognitive control processes in children with ADHD. In addition, decreased PerAF values in the left cerebellar lobule VIII at baseline may indicate reduced local spontaneous activity in a cerebellar region involved in motor planning and cognitive regulation. This interpretation aligns with prior ADHD studies reporting altered local brain activity using other voxel‐based rs‐fMRI metrics such as ReHo and degree centrality (Wang et al. 2017). Consistent with this interpretation, Rubia et al. reported reduced cerebellar activation and weakened cerebellar–cortical functional connectivity in children with ADHD during cognitive tasks, supporting the involvement of the cerebellum in network‐level dysfunctions associated with the disorder (Rubia et al. 2009).

Following VR intervention, decreased PerAF values in the right MFG may indicate improved neural efficiency or reduced compensatory load within cognitive control networks, which was accompanied by behavioral improvements in hyperactivity and impulsivity. Pharmacological studies have shown that effective treatment can normalize excessive frontal cortical activation in individuals with ADHD (Wang et al. 2025). This study observed a similar phenomenon in a non‐pharmacological VR intervention, providing new imaging evidence for its mechanism of action. In parallel, the increased activity observed in the left PCL is of particular functional relevance. The PCL serves as a critical hub for sensorimotor integration, linking primary motor and somatosensory cortices (Zhang et al. 2020). Previous studies have reported reduced structural connectivity between the right cerebellar lobule VI and adjacent cortical regions, including the left PCL, in children with ADHD (Liang et al. 2023). Enhanced PCL activity following VR intervention may reflect strengthened online integration of sensory input and motor output through repeated, embodied task engagement, thereby contributing to improved behavioral inhibition and motor control precision.

It is worth noting that, after VR intervention, the attention deficit scores of children with ADHD and the high activity of the left ITG still significantly differed from those of typically developing children. This finding warrants in‐depth exploration. There are several possible explanations for the post‐intervention persistence of higher left ITG activity. First, it is possible that this region reflects a residual disorder‐related abnormality not substantially modulated by the current training protocol. Second, increased post‐intervention ITG activity may reflect a compensatory neurocognitive strategy. Such a mechanism could involve greater reliance on visual‐semantic or emotionally salient cue processing during behavioral regulation. This interpretation is consistent with the ITG's role as a key interface between the ventral visual stream and the limbic system, as it is deeply involved in complex visual processing, emotional face recognition, and emotion regulation (Li and Kong 2017; Xie et al. 2022). Persistent hyperactivity in this region may reflect difficulties in underlying deficits in emotional regulation. Third, the lack of significant changes in ITG activity could highlight task‐specific limitations of the current VR program. The modules were primarily designed to focus on response inhibition and dynamic attentional engagement and therefore may not have sufficiently targeted temporal‐limbic processes underpinning higher‐order cognitive‐emotional integration. Previous studies, including work by Su, have provided functional connectivity evidence supporting the role of ITG in emotional processing (Su et al. 2015). The observed positive correlation between ITG activity and oppositional defiant symptoms further supports this interpretation. These findings suggest that VR tasks primarily focused on response inhibition and dynamic attentional engagement may have limited effects on temporal‐limbic circuits involved in higher‐order cognitive‐emotional integration. Therefore, the specific design of VR tasks still needs to be further improved.

Although sustained attention was one of the intended training targets, the present study did not observe a robust improvement in parent‐rated inattention symptoms. Several potential explanations for this finding can be proposed. One possible explanation is that the current VR program may have had a more pronounced impact on behavioral inhibition and overt hyperactive/impulsive control, while its effects on the core inattentive dimension were less significant. This could indicate that different symptom dimensions of ADHD might require more targeted intervention approaches. Another explanation could lie in the limitations of the SNAP‐IV, which might lack the sensitivity needed to detect subtle, short‐term cognitive changes. In contrast, the MFFT may be better suited for capturing specific improvements in attentional performance (Viator et al. 2022). A further possibility is that the lack of generalization might be due to the relatively short duration of the intervention. Future research is essential to evaluate these potential explanations more thoroughly.

From the perspective of regional intrinsic brain activity, this study provides preliminary evidence identifying potential neural targets of VR intervention in ADHD, thereby informing the development of more targeted digital therapeutics. Future studies integrating functional connectivity or network‐level analyses may further elucidate the neural mechanisms underlying VR‐based interventions from a systems neuroscience perspective.

Several limitations should be acknowledged. First, the relatively small sample size limits the generalizability of the findings and warrants replication in larger cohorts. The small sample size limits sensitivity to detect small‐to‐moderate effects and increases the risk of both false‐negative findings and unstable effect‐size estimates. Our study should be regarded as exploratory. Second, the absence of an active control group precludes definitive conclusions regarding the specificity of VR effects. Non‐specific factors, such as repeated testing, increased attention from researchers, novelty effects, and the structured weekly intervention schedule, may also have contributed. Accordingly, the current findings should be interpreted as preliminary, and future randomized controlled studies including both active and non‐intervention comparison groups are needed to determine the specificity of VR‐related effects. The current findings should be interpreted as preliminary evidence supporting the feasibility and potential neural correlates of VR‐based training rather than definitive proof of VR‐specific therapeutic mechanisms. Future randomized controlled studies with active comparison conditions will be necessary to address this issue rigorously. Third, the short follow‐up period prevents assessment of the long‐term stability of behavioral and neural changes. Future studies should incorporate more refined VR task modules specifically targeting sustained attention or emotional regulation, employ randomized controlled designs with larger samples, and include extended follow‐up periods to comprehensively evaluate the long‐term impact of VR intervention on neurodevelopmental trajectories in ADHD. Finally, the inclusion of treatment‐naive children (aged 6–14) in our study is a notable strength, as it minimizes the confounding effects of prior medication on brain activity. However, it may introduce selection bias, as this sample may represent children with milder symptoms or those from families facing barriers to accessing medication. To enhance the generalizability of findings, future research should include ADHD populations with prior medication history and explore their responses to VR‐based interventions in comparison with treatment‐naive participants.

## 5 Conclusion

In conclusion, VR intervention may improve hyperactivity/impulsivity and behavioral symptoms in children with ADHD by facilitating normalization of frontal regions involved in executive control, such as the right middle frontal gyrus, and by enhancing activity in regions supporting sensorimotor integration, including the left PCL. However, VR‐specific effects cannot be isolated in the absence of an active control condition. As a promising adjunctive intervention, future clinical translation of VR‐based approaches for ADHD may benefit from symptom‐domain‐specific optimization, an active longitudinal control group, and individualized training strategies informed by neurobiological evidence.

## Author Contributions

**Xinjie Yu**: writing – original draft, writing – review and editing. **Jieling Zhu**: conceptualization, methodology. **Jiujiu Yang**: methodology, software. **Hongtao Hou**: data curation, investigation. **Guoqun Mao**: validation, formal analysis. **Luhan Tang**: supervision, funding acquisition. **Fuquan Wei**: writing – review and editing, writing – original draft.

## Funding

This research was supported by the TCM Science and Technology Program of Zhejiang Province, China (Grant No. 2023ZL030 Fuquan Wei), the Medical and Health Science and Technology Plan Project of Zhejiang Province (Grant No.2024KY876, 2025HY0251; Fuquan Wei) and State Administration of Traditional Chinese Medicine Science and Technology Department‐Zhejiang Provincial Administration of Traditional Chinese Medicine Co‐construction of Key Laboratory of Research on Prevention and Treatment for depression syndrome[NO. GZY‐ZJ‐SY‐2402].

## Ethics Statement

This study was conducted in accordance with the approved protocol of the institutional review board with the assigned approval number Zhe Tong De lun Review 2023 Yan 013‐JY.

## Conflicts of Interest

The authors declare no conflicts of interest.

## Data Availability

The data that support the findings of this study are available on request from the corresponding author. The data are not publicly available due to privacy or ethical restrictions.
